# Impact of Mediterranean Diet on Chronic Non-Communicable Diseases and Longevity

**DOI:** 10.3390/nu13062028

**Published:** 2021-06-12

**Authors:** Ligia J. Dominguez, Giovanna Di Bella, Nicola Veronese, Mario Barbagallo

**Affiliations:** Geriatric Unit, Department of Medicine, University of Palermo, 90127 Palermo, Italy; ligia.dominguez@unipa.it (L.J.D.); giovannadibella79@libero.it (G.D.B.); nicola.veronese@unipa.it (N.V.)

**Keywords:** aging, diet, lifestyle, longevity, cardiovascular, cancer, chronic, dementia, Mediterranean diet, mortality

## Abstract

The average life expectancy of the world population has increased remarkably in the past 150 years and it is still increasing. A long life is a dream of humans since the beginning of time but also a dream is to live it in good physical and mental condition. Nutrition research has focused on recent decades more on food combination patterns than on individual foods/nutrients due to the possible synergistic/antagonistic effects of the components in a dietary model. Various dietary patterns have been associated with health benefits, but the largest body of evidence in the literature is attributable to the traditional dietary habits and lifestyle followed by populations from the Mediterranean region. After the Seven Countries Study, many prospective observational studies and trials in diverse populations reinforced the beneficial effects associated with a higher adherence to the Mediterranean diet in reference to the prevention/management of age-associated non-communicable diseases, such as cardiovascular and metabolic diseases, neurodegenerative diseases, cancer, depression, respiratory diseases, and fragility fractures. In addition, the Mediterranean diet is ecologically sustainable. Therefore, this immaterial world heritage constitutes a healthy way of eating and living respecting the environment.

## 1. Introduction

A demographic revolution has happened in the past 150 years for the first time in history. At the end of the nineteenth century, life expectancy was near 35 years in Europe similar to that in the Roman Empire of about 30 years [1]. At present, numerous citizens from most European countries and Japan live to be over 80. The World Report on Ageing and Health published by the World Health Organization (WHO), confirms that currently most people worldwide can anticipate living to be over 60 [2].

The decline in mortality from infectious diseases was probably a major contributor to this astonishing demographic event. Nevertheless, child mortality decline was not the single cause of greater life expectancy, because mortality rates changed also in adults older than 50 years [3]. The reduction of infectious diseases has paralleled an increase in age-associated diseases, including cardiovascular disease (CVD), neurodegenerative diseases, and cancer, so-called non-communicable diseases (NCDs) [4,5]. A longer living human being has an extended time of exposure to risk factors, which increases the possibility of disease. This phenomenon is occurring not only in developed countries but also in developing and underdeveloped world regions [6].

In this context, morbidity and mortality linked to NCDs have grown progressively with an expected increase in the number of people unable to perform basic daily activities and worsening quality of life [1,4,7,8]. Because NCDs are more commonly observed in aging populations, the phenomenon of world aging is a chief factor explaining the increase observed in these diseases. The repercussion for health personnel, health systems and finances are profound [2,8,9]. The endeavors of medicine should aim not merely at life’s prolongation; instead, at searching ways of promoting old age eluding multimorbidity and disability as far as possible.

Unfavorable lifestyles, which are commonplace today and involve significant proportions of the population, have been identified as a primary contributor to the greater incidence of risk factors [7,9]. Reduced physical activity and sedentary lifestyle, unhealthy diet, excess eating, and smoking are crucial determinants of the rise of obesity, type 2 diabetes (T2D), hypertension, and lipid profile alterations, all strong risk factors for CVD, dementia, and some forms of cancer [7,8,10,11,12,13].

In recent decades, nutrition research attention has switched from studying the effects of single nutrients and foods to studying dietary patterns, assuming that combinations of foods/nutrients can have synergistic and/or antagonistic actions beyond individual components [14]. In this context, various dietary patterns have been associated with health benefits [12]. For example, the traditional dietary pattern and way of living followed by population from Mediterranean countries (Mediterranean diet, MedDiet), may help preventing chronic diseases and premature mortality [15,16,17]. MedDiet was designated in the 2015–2020 Dietary Guidelines for Americans among the healthiest dietary patterns recommended [18,19]. The MedDiet has been associated with better nutrient sufficiency in diverse observational and intervention studies aiming to evaluate deficits of fiber, calcium, potassium, and magnesium, all dietary elements of public health relevance [20]. This dietary pattern, mostly based on plant-derived foods (but also admitting animal-derived food in small quantities), preferring seasonal and local food consumption and production, constitutes an eating pattern that considers both health and environmental issues. The traditional MedDiet is not only a group of healthy foods, but also a cultural archetype that comprises the manner in which foods are selected, processed, and dispensed, together with other foundations of lifestyle. These characteristics led UNESCO in 2010 to include MedDiet on the list of the intangible cultural heritage of humanity [21].

Regrettably, diets currently followed by many Mediterranean populations are dissimilar to the traditional MedDiet, particularly when it comes to portion sizes, proportions of food groups, and use of industrial food [22]. This is particularly true for children and adolescents [23], due in large part to the dissemination of Western eating habits and globalization of food production, which has homogenized world eating behavior in modern times.

This narrative review aims to examine the course of MedDiet as it was described initially in the 1950s up to now, and to underline the evidence and diverse perspectives that have emerged in the past seven decades concerning the relationship between this MedDiet pattern and the decreased mortality and multimorbidity derived from age-associated NCDs. We first describe the definition and history of MedDiet, proposed mechanisms to explain its beneficial effects, and methods of assessment of adherence to MedDiet. We then present the main studies showing associations of a higher adherence to MedDiet and diverse NCDs, comprising CVD, cognitive decline and depression, cancer, metabolic diseases, chronic respiratory disease, and bone fragility, as well as studies reporting associations of MedDiet and mortality. Lastly, we discuss the relationship of MedDiet, sustainability, and the extrapolation of MedDiet to non-Mediterranean countries.

## 2. What Is ‘Mediterranean Diet’ and Where Does It Come from

The notion of MedDiet, as it is conceived today in nutritional research, was first introduced by the Seven Countries Study in the 1950s, when the globalization had not started to guide lifestyle and diet. In this seminal study, Southern European populations from countries where olive-trees grow naturally, exhibited longevity among the highest in the world, with the lowest incidence of coronary heart disease (CHD), cancer, and other NCDs [24]. These populations did not follow a specific dietary pattern, but some traditional eating and lifestyle habits, originated centuries before, and remaining similar in identical locations. This persistence has aroused interest among historians, physicians, gastronomes, and agronomists, among other scholars.

**Figure 1:**
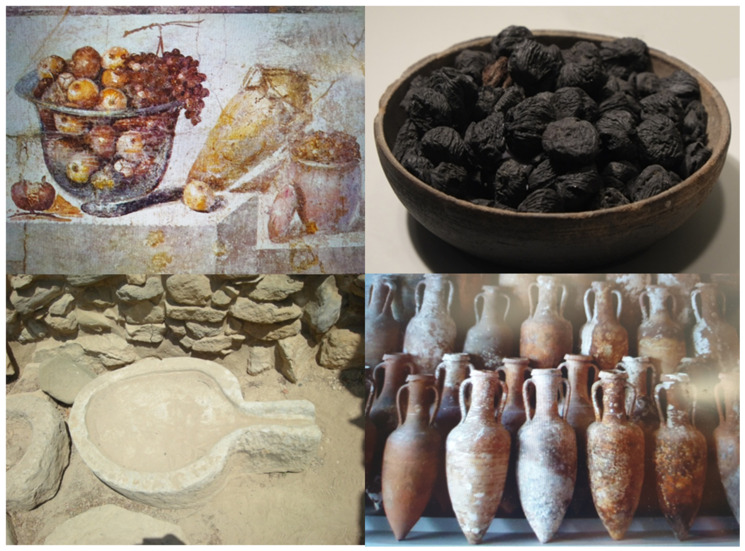
Photographs of archaeological vestiges illustrating the persistence of MedDiet for centuries since ancient times. Clockwise from top left corner, still life with glass fruit bowl and vases, House of Julia Felix, Pompeii, Italy; whole dried figs (*Ficus carica* L.) charred, from Pompeii, National Archaeological Museum of Naples, Italy; traditional olive oil jars, “Luigi Bernabò Brea” Aeolian Regional Archaeological Museum, island of Lipari in Sicily, Italy; traditional olive oil press, Crete, Greece.

Through history, societies have created a myriad of foods combinations from those available in their specific areas of residence, resulting in the generation of the traditional dietary patterns. Some of them, e.g., MedDiet, the Japanese and the vegetarian diets, have shown beneficial associations with diverse health outcomes [25]. The term MedDiet, in addition to its nutritional value, has a profound emblematic significance linked to a distinctive lifestyle in the Mediterranean basin, the cradle of Western civilization. Historical, traditional, and literary sources confirm the persistence of the traditional Mediterranean eating habits, comprising archaeological relics, such as tombs’ food leftovers or charred foods found from the eruption of Mount Vesuvius in 79 a.d., artefacts used for manufacturing, eating, and cooking, and containers for transporting food. Additionally, there are classical literary sources from Homer on, terracotta tablets, frescoes, and papyri, among others [26] (Figure 1).

The main generic characteristic of what is called today MedDiet is its plant-based composition. It encompasses consumption of abundant seasonal vegetables, olive oil for cooking or seasoning (main fat source); fresh seasonal fruit consumed as a desert; regular consumption of nuts and seeds (either as part of the recipes or as healthy snacks); consumption of legumes several times weekly; whole cereals daily; consumption of moderate portions of fish two to three times weekly; dairy (yogurt, milk, cheese) several times per week in limited amounts; spices and herbs to flavor recipes; infrequent consumption of sweets (a few times weekly); inclusion of red and processed meat with utmost moderation in small amounts; three to four eggs weekly; use plenty of water as beverage; drinking wine in moderation, always with meals and respecting each community beliefs [27]. A hallmark of MedDiet is the inclusion of unprocessed foods, which are full of healthy nutrients, as opposed to Western dietary patterns, which are rich in processed and ultra-processed foods, full of calories but very poor in nutrients (“empty calories”), linked to a high risk of developing overweight/obesity [28,29].

MedDiet comprises dietary choices but also lifestyle, as well as historical knowledge, traditions, abilities, and practices, which have been passed down through the generations, extending from the countryside and food production to the ways of cooking, which provide a feeling of belonging and permanency to the community. Mediterranean traditional cuisines are rich in aromas, colors, and memories, highlighting the taste and the synchronization with nature, and emphasizing the significance of preparing and consuming foods jointly with family and friends. The traditional MedDiet was shaped by the environment, the flora, as well as by hardship [21]. The production of food at the time of first MedDiet description by Ancel Keys was not industrial and involved a conspicuous physical effort, which is crucial in the context of the multiple benefits derived from combining dietary with lifestyle factors [30] (Table 1).

The history of how the name ‘Mediterranean Diet’ originated, started in 1948, when the Greek government, concerned about the difficult economic, health and social conditions in the post-war period, invited the Rockefeller Foundation to perform a population study on the way Cretan dwellers used to eat. Crete was a place where traditions had not changed for centuries. Leland G. Allbaugh headed a group of American epidemiologists interviewing a random sample of the population, to obtain information on dietary and lifestyle parameters. Plant-foods provided about 61% of the energy consumed, much higher than the 37% recognized in the USA at that time. The unexpected finding was that people from Crete had a diet with a fat content that was not different from that of Americans (~100 g/day), but the main source was olive oil for Cretans vs. animal origin fat (saturated fatty acids: SFA) for the American diet. Surprisingly, this and other observations of the Cretan diet in the 1950s and 1960s were associated with excellent health standards, despite not yet receiving the “advances” of the Western industrialized world. In fact, the researchers were startled because they had assumed beforehand that not being exposed to “civilization” could have provoked harmful effects on this population due to their poor food, and low social, and sanitation conditions. Ancel Keys learned about these results of Allbaugh et al. and became interested in the argument traveling as a visiting researcher to Southern Italy. The concept of the MedDiet originated from these reflections, leading Ancel Keys et al. to coin the term and to planning of the Seven Countries Study [24]. Indeed, Ancel Keys and his wife Margaret introduced the name Mediterranean diet into the Western lexicon with their popular book “How to eat well and stay well the Mediterranean way”. This term officially entered into the scientific literature thanks to research on the relationship of diet and cholesterol, strongly backed by Ancel Keys, conducted by Anna Ferro-Luzzi et al. in Cilento, Italy [31].

At the time of the Seven Countries Study, Cretan farmers were disappointed and used to complain in front of the interviewers about how poor was their diet with so little meat, ignoring the fact that probably that was one of the main explanations for their outstanding health, in combination with their heavy labor in the rural areas. In fact, the concept that excess food is associated with unhealthy consequences was usually not considered in times of scarcity, when fatness could be seen as a representation of good health. At that time, people would hardly believe a physician’s advice on the benefits of a parsimonious diet, composed of foods considered “poor” with a low intake of meat and sweets, in combination with constant physical effort that was seen as mortifying. This type of diet and lifestyle, which in the future would be acknowledged as one of the world’s most healthy diets, was the result of consuming food that was available at the limit of subsistence, where a frugal diet composed of products provided by nature was a necessity more than a life choice. This probably helps to explain why once these societies were in contact with the economic boom at the end of the twentieth century, they tended to abandon this way of eating and living preferring the indulgences of the Western model of diet and lifestyle. The difficulty in transmitting messages of prevention today is not far from these past testimonies. It is challenging to convince the lay public that they should eat sparingly and move constantly when they eagerly search for an indulgent lifestyle asserting that they can afford to live with little physical exercise and overeating [32,33].

In summary, the MedDiet pattern consists of choosing healthy foods, in the background of ancient traditions deep-rooted in the historical and cultural characteristics of rural societies that for centuries have followed them without being aware of the immense benefits this entailed. This diet is not just associated with great health benefits, but it is also tasty, which can facilitate adherence for a longer time, adopting it as a usual eating model and lifestyle [34].

## 3. What Mechanisms May Help Explain MedDiet Benefits

The mechanisms explaining the multiple observed benefits of adhering to the MedDiet have not been fully elucidated. However, several molecular and clinical pathways have been intensely studied suggesting plausible beneficial modifications induced by this dietary pattern. Some of the proposed mechanisms are common to different pathological conditions, such as the reduction of oxidative stress and chronic inflammation mainly attributable to bioactive antioxidant components of foods that are part of MedDiet (Table 2, Table 3 and Table 4). As shown below, most of these investigations were carried out in experimental studies with isolated compounds, but also there is some evidence from epidemiological studies testing the associations of health outcomes with the dietary inflammatory index (DII) [35] or analyzing the dietary content of polyphenols in FFQ from longitudinal cohorts. Hodge et al. examined associations of DII and MedDiet with total, CVD and CHD mortality in the Melbourne Collaborative Cohort Study. A higher score of adherence to MedDiet and lower DII were associated with lower total, CVD and CHD mortality, with similar power of the associations for both scores using the Bayesian information criterion [36]. Analyses from the ‘Seguimiento Universidad de Navarra’ (SUN) longitudinal cohort reported that the risk of CV events progressively increased with each increasing quartile of DII; in the multivariable-adjusted model, HR for participants in the highest vs. the lowest quartile of the DII was 2.03 (95% CI 1.06–3.88) [37]. Data from the PREDIMED (prevención con dieta mediterránea) trial, the SUN cohort and from a random-effect meta-analysis of 12 prospective studies showed that the highest vs. the lowest quartiles of DII were positively and significantly association with all-cause mortality in both the SUN and the PREDIMED cohort, as well as in the meta-analysis of 12 cohorts [38]. A systematic review of observational and experimental research assessing the effects of polyphenols found in a MedDiet on depression symptoms found an association between polyphenol consumption and depression risk, as well as evidence suggesting that polyphenols can effectively alleviate depressive symptoms [39]. Analyses from 21,302 participants of the Mediterranean Moli-sani cohort followed for a mean of 8.3 years showed that those in the highest quintile of intake of various polyphenols presented a significant lower all-cause mortality risk vs. those in the lowest group of consumption [40]. In this population, multiple regression analyses showed that the polyphenol antioxidant content (PAC) score and its seven components were positively associated with MedDiet adherence in both genders with one unit increase in PAC score being associated with 7.1–7.8% increase in the likelihood of high MedDiet adherence [41]. In a Mediterranean cohort from Catania (Italy) the mean intake of polyphenols was high (663.7 mg/d) and major food sources of polyphenols were nuts, followed by tea and coffee as sources of flavanols and hydroxycinnamic acids, respectively, fruit (i.e., cherries were sources of anthocyanins and citrus fruit of flavanones) and vegetables (i.e., artichokes and olives were sources of flavones and spinach and beans of flavanols); and chocolate, red wine and pasta contributed to flavanols and tyrosols, respectively [42]. An RCT compared the effects of a high-polyphenol diet with six portions of fruit and vegetable daily vs. less than two portions on microvascular function in hypertensive participants. After eight weeks, endothelial function was better for participants on the high-polyphenol diet, which was related to higher levels of serum polyphenols [43]. In the PREDIMED trial, a high intake of total polyphenols, total flavonoids (specifically flavanones and dihydroflavonols), and stilbenes was associated with a reduced risk of diabetes in elderly persons at high risk of CVD [44]. In the same trial, those who reported a high-polyphenol intake, especially of stilbenes and lignans, showed a reduced risk of overall mortality compared to those with lower intakes [45], with the main dietary source of polyphenols in PREDIMED participants being coffee and fruit, and the most important differentiating factor with respect to other populations was the consumption of polyphenols from olives and olive oil [46].

In addition, there are some proposed mechanisms that are disease-specific (e.g., cancer, CVD) such as changes in hormones and growth factors linked to cancer pathogenesis [47,48]. Women following a MedDiet had a significant elevation of the plasma concentration of several binding proteins, such as insulin-like growth factor binding protein (IGFBP)-1, IGFBP-2, and sex hormone binding globulin, resulting in a reduction of the biological activity of IGF-1, testosterone, and oestradiol [47]. Insulin, estrogens, androgens, and IGF-1 are powerful cellular mitogens, linked to the development and growth of several tumors, including breast, colon, prostate, pancreatic, and endometrial cancer [48]. Additionally, specific amino acid limitation may lead to inhibition of some nutrient sensing pathways [49]. The MedDiet typically has a lower content of animal proteins compared to the Western diet. This is important because protein restriction has been linked to life span extension, independently of energy intake, in multiple experimental models [50]. Regarding CVD, several studies showed the effects on lipid metabolism [51] and platelet aggregation [52] towards cardio-protective profiles associated with MedDiet, as well as improved endothelial function at different vascular beds [53].

Other proposed pathways to explain the impact of dietary factors on aging and on incident NCDs are epigenetic mechanisms, such as DNA and histone methylation, histone acetylation, as well as non-coding RNA [54]. As opposed to what happens to DNA that cannot change, epigenetic modifications can occur in response to various stimuli, such as the exposure to nutrients, toxins, pesticides, and pollutants. Interestingly, various MedDiet components comprise bioactive elements that have been lately studied, particularly for their antitumor actions [55].

The polyphenolic compounds, e.g., those found in grapes, berries, peanuts, extra-virgin olive oil (EVOO), etc., have been associated with DNA methylation of various genes linked to cancer, as well as of crucial tumor-suppressors and promoters [56]. Anthocyanins, pigments contained in berries, eggplants, black grapes, pomegranate, and cruciferous vegetables, were shown in vitro to impact the cell cycle by epigenetic modifications, stimulating DNA repair mechanisms [57]. Fisetin is a flavonoid contained in apples, cucumbers, strawberries, onions, and persimmons, which has been reported to prevent cancer cells growth altering various signaling pathways, such as cell division, angiogenesis, metastasis, oxidative stress, and inflammation [58]. Sulforaphane contained in cruciferous vegetables is a Sulphur-rich compound displaying recognized epigenetic actions via histone deacetylase enzymes inhibition [59]. The carotenoid lycopene with antioxidant properties, contained in tomatoes at high concentrations and at lower concentrations in many other fruits, has been linked to reduced prostate cancer risk by the regulation of serine/threonine kinase 2 and MicroRNA-let-7f-1 expression [60]. SAMP8 mice, an experimental model of accelerated aging and learning/memory impairment, which received EVOO and polyphenols from EVOO had improvements in memory and learning tests when compared to mice receiving butter, in parallel with improved indexes of oxidative stress [61]. Quercetin, a flavonoid contained in berries, cruciferous vegetables, red grapes, red onion, tomatoes, and citrus fruit, were associated with the repression of tyrosine kinase janus kinase 2, shown to induce apoptosis and autophagy in cancer cells [62]. In a subgroup of 36 participants of the PREDIMED-Navarra study, epigenetic modifications of genes linked to immunocompetence and inflammation were observed in peripheral blood cells, which were correlated with the degree of adherence to MedDiet [63]. EVOO and nuts, hallmarks of MedDiet, were reported to induce methylation in several genes from peripheral leukocytes [64]. Preliminary results from the NU-AGE project, involving 120 healthy older adults, showed that adhering to a MedDiet-like pattern for one year was associated with epigenetic modifications [65]. Nevertheless, the above-mentioned studies are promising but still experimental, mainly carried out in cells and experimental animals. Further research is necessary to clarify whether epigenetic mechanisms exerted by these bioactive compounds truly help to elucidate some of the benefits of MedDiet.

In fact, MedDiet is composed of a combination of biologically active foods that renders unique this dietary model [66], i.e., a proper mixture of salutary sources of fats, starches, proteins, fiber, minerals, vitamins, and a myriad of bioactive components including phytosterols, terpenes, and polyphenols, and other yet undisclosed compounds, which may help to elucidate its various benefits. Jointly, they can probably act synergistically by means of various biological and molecular mechanisms to lessen the risk of NCDs, hence influencing morbidity and mortality (Table 2, Table 3 and Table 4).

**Figure 2:**
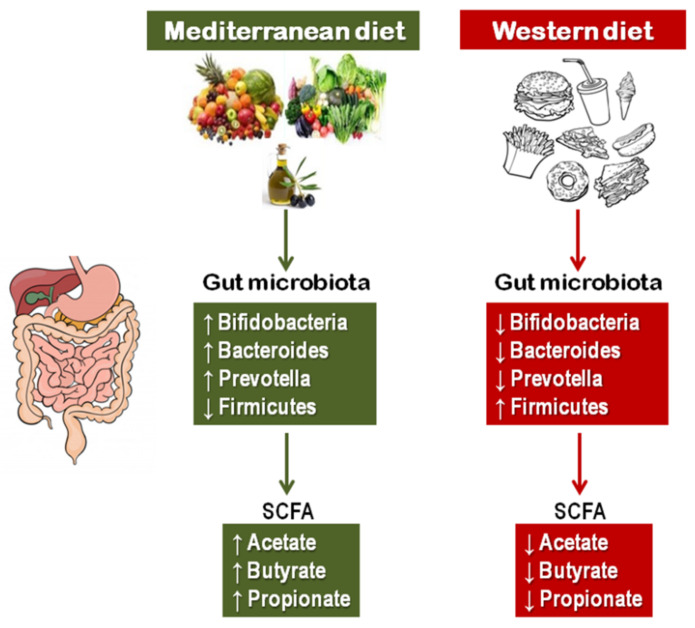
The MedDiet and the Western diet exert opposite effects on the composition of gut microbiota with consequent modifications in SCFA production.

A key mechanism that can help explain the benefits of MedDiet is the gut microbiota, which in recent decades has emerged as a crucial player in the relationship between diet and health by means of metabolites derived from the microbial fermentation of nutrients, particularly short chain fatty acids (SCFAs) (Figure 2). In fact, diet is a major regulator of gut microbiota composition and metabolite production, which has been related to the incidence and progression of several intestinal and extra-intestinal diseases [67,68,69]. There are some studies examining specifically the effects of the whole MedDiet pattern on microbiota composition indicating that the MedDiet elicits favorable microbiota profiles and SCFAs production, with microbial diversity resembling adherence to this dietary pattern [70,71,72]. A high adherence to the MedDiet was associated with an enrichment of Firmicutes and Bacteroidetes and an increase in fecal SCFAs. Conversely, poor adherence to MedDiet was associated with increased l-*Ruminococcus* and Streptococcus bacteria, and higher urinary trimethylamine N-oxide concentrations, a marker of increased risk of CVD [70]. A prospective study in 912 patients with atrial fibrillation treated with vitamin K antagonists reported that levels of gut-derived lipopolysaccharide (LPS) were predictive of major adverse CV events and negatively associated with high adherence to MedDiet [71]. A study in 27 healthy volunteers showed that a better adherence to MedDiet was associated with significantly higher level of total SCFAs [72]. Another study in 23 overweight omnivores comparing the MedDiet with a vegetarian diet reported a significant increase in propionic acid production that was negatively correlated with changes in various inflammatory cytokines only in participants following the MedDiet [73]. A recent investigation of 360 Spanish adults from the Obekit cohort reported that higher adherence to MedDiet, as well as fiber, legumes, vegetables, fruit, and nut intakes were associated with an increase in butyrate-producing taxa [74]. A hallmark of MedDiet is its high content of fiber [75]. It has been shown that high dietary fiber intake promotes modifications of gut microbiota in both rodents and humans, with decreased Firmicutes and increased Bacteroidetes, which produces high levels of SCFAs, related with several inflammatory, autoimmune, and allergic diseases [76].

## 4. Assessment of Adherence to the MedDiet

The Seven Countries Study was fundamentally an ecological investigation. Later on, the need for an operational definition that would allow comparisons between epidemiological studies in different populations assessing the effects on health outcomes linked to the conformity with the MedDiet was apparent. The first and most frequently used MedDiet score was developed by Trichopoulou et al. in 1995 through a simple score [77], which was afterwards updated [78]. In brief, Trichopoulou’s MedDiet score allocates one point for the consumption of each component with potential health benefits (vegetables, legumes, fruit and nuts, fish, cereals, and the ratio of monounsaturated fatty acids (MUFA) to SFA); the cut-off points considered are the sex-specific medians of consumption for each component within the studied population. For potentially unfavorable components (meats, processed meats, dairy products—seldom low-fat in the Mediterranean region), a consumption that is below the sex-specific median for the studied population is counted for by adding one point to the total score; for higher amounts the value is zero with no added points. For alcohol, one point is added to the score for men consuming 10 to 50 g per day and for women consuming 5 to 25 g per day. Thus, the total score ranges from zero (lowest adherence to MedDiet) up to 9 (highest adherence) [78]. The fact that this score is highly reliant on the specific sample characteristics (based on the sample medians) can be a limitation for the appraisal of results from different samples and populations, and in particular, for the transferability of the findings among diverse populations. Alternatively, another score was developed for the PREDIMED intervention trial, called the Mediterranean Diet Adherence Screener (MEDAS) [79,80]. This score uses absolute/normative cut-off points (pre-defined daily or weekly consumed servings) for specific food groups [80,81,82]. The advantage of MEDAS is its comparability between different studies and populations because it does not depend on the characteristics of the samples. Additionally, it can be used directly, in an immediate way, for the evaluation of the conformity with the MedDiet as an instrument to encourage people to improve their adherence to this dietary pattern. Table 5 describes foods and assigned points used by MedDiet score and by MEDAS. In a study evaluating prospectively the association of adherence to MedDiet with a composite outcome of incident CV events, incident T2D or all-cause mortality comparing Trichopoulou’s MedDiet score and MEDAS, both scores similarly predicted the distribution of macronutrients and the disease incidence or mortality [82].

Several other scores have been proposed aiming to ascertain the adherence to MedDiet. As expected, there are some disparities among the scores possibly due to many reasons, not yet fully clear. The disagreements may depend, at least in part, on the dissimilar geography, historical time, and authors’ nationality; these regions may have different dietary patterns and lifestyles despite some common features that support the compelling evidence of the MedDiet’s benefits. A systematic review evaluated 70 original studies using MedDiet scores for diverse health outcomes [83]. Almost half of the studies used Trichopoulou’s MedDiet score, 14 studies using the original version and 18 studies using nine different versions with some variations of the original Trichopoulou’s definition. Another proposed score by Fung, et al. was used by additional 14 studies in the original form or with small variations of it. The rest of the scores were used less frequently. Nevertheless, all the studies were in agreement with the protective role of MedDiet against multiple health outcomes [83].

## 5. Search Strategy

PubMed (Medline) and Scopus were searched for observational and intervention studies as well as systematic review with or without meta-analysis, published in English, Italian or Spanish and present in peer-reviewed journals between the databases’ inceptions and 1 March 2021 that examined the association between Mediterranean diet and health outcomes. The titles and abstracts were independently screened by three of the investigators (LJD, NV, and GDB). The full texts of potentially relevant papers were examined by three of the investigators (LJD, NV, and GDB), and discrepancies were resolved through discussion with a senior author (MB).

## 6. Effects of MedDiet on Incident NCDs and Longevity

Considering the persistent rise in older adults among world populations with the predictable concern that health systems can turn out to be unsustainable if numerous seniors have multi-morbidity and disabilities, there is raising awareness of the indisputable necessity of implementing programs for the primary and secondary NCDs prevention. In fact, hypertension, CHD, diabetes, cerebrovascular diseases, osteoarthritis, and cancer at present are responsible for most of world’s health care expenditures [2]. Compelling knowledge in nutritional research suggests that increasing adherence to MedDiet pattern is associated with a reduction in total and cause-specific mortality [24,77,78,84,85,86,87,88,89]. Furthermore, a lower incidence of major NCDs in older adults, including CVD [15,24,79,90,91,92,93,94,95], metabolic syndrome, obesity, and diabetes [96,97,98,99,100,101,102,103,104], some types of cancer [105,106,107,108,109,110,111,112], cognitive decline and dementia [90,113,114,115], and unipolar depression [116], are associated with lifelong healthy dietary practices, such as those of the MedDiet pattern. There is also evidence for a decrease in major CV risk factors, including hypertension, inflammatory markers, and dyslipidaemia [117,118,119]. The beneficial effects of MedDiet seem to be linked to the interactive integration of foods and nutrients more than to their isolated actions. Thus, nutritional factors can considerably impact older adults’ health and functional status, but moreover a lifetime approach for the prevention of NCDs, it is crucial to recognize risk factors for malnutrition, which are frequently observed in old age [120].

### 6.1. Cardiovascular Disease

Despite several modifiable risk factors for CVD, such as an unhealthy diet, smoking, obesity, and sedentary lifestyle, have been identified a long time ago, CVD continues to be the leading mortality cause in Western countries [121]. Hence, a public health priority is certainly promoting and preserving CV health through the modifications in lifestyle. A key finding of the seminal Seven Countries Study [24], was that the Cretan population had about 30 times lower CHD rates when compared to citizens from Finland, despite both populations had similar fat consumptions (36 to 39% of the total energy intake). Yet SFA accounted for ~24% of energy intake in Finish participants vs. ~8% in Cretans. The type of fat in EVOO (mainly MUFA) and omega-3 PUFA contained in fish and nuts, now recognized as cardio-protective, could have contributed, at least in part, to the lower incidence of CV events in participants from Crete. Noteworthy, in the EPIC study comparing the content of MUFA in different countries, the western diet and the MedDiet had not quite different amounts of energy intake derived from MUFA (i.e., 14.5–20.8% in Greece, Spain and Italy vs. 13% in Germany and Sweden); however, MUFA in MedDiet comes mainly from the high consumption of EVOO, while in the Western diet MUFA are mainly supplied from animal foods, such as red meat and whole dairy products [122]. When the Seven Countries Study was conducted, the lower incidence of CHD with MedDiet was attributable to the decrease in blood cholesterol; however, later investigations confirmed that MedDiet is not just a diet able to lower cholesterol, but that it has various added health benefits, as discussed below.

The first randomized trial (RCT) reporting a robust CVD protection associated with a dietary intervention was the Lyon Diet Heart Study [123]. This was a secondary prevention study, recruiting 605 patients with a history of myocardial infarction and randomly assigned to a MedDiet pattern vs. a low-fat diet recommended by the American Heart Association. Participants in the intervention group were encouraged to increment the consumption of vegetables, fish, and fruit, while reducing the consumption of red meats. In addition, they received advice to substitute butter and cream with a margarine rich in linolenic acid, an omega-3 PUFA. The results were striking, showing a 73% relative reduction in the recurrence of CHD events after a mean follow-up of 27 months. Because in this study EVOO was not used, instead an omega-3 PUFA rich substitute, adjustments of the original description of MedDiet were developed, which were more appropriate for non-Mediterranean populations. In the USA, consumption of omega-3 PUFA in 2005–2006 contributed about 0.05% of the energy intake [124], while in the PREDIMED trial the baseline consumption of omega-3 PUFA was 0.32%, about six fold higher [79]. Therefore, significant contrasts of MedDiet and the USA diet lay not only in the sources and amount of MUFA, but in the amount of omega-3 PUFA as well.

The PREDIMED intervention trial [79] showed that following a MedDiet pattern, supplemented with EVOO or nuts, with a fairly high fat intake (35–40% of the total energy intake), decreased incident major CV events by near 30% in participants with high CV risk over 4.8 years of follow-up when compared to a control group allocated to a low-fat diet, defined as a fat intake of ≤30% of total energy intake according to the food frequency questionnaire (FFQ). The MedDiet score increased in both intervention groups and stayed unaffected in the low-fat group. The trial was terminated early due to the positive results, which met the benefit requirements.

Numerous prospective cohort studies and meta-analyses showed significant associations of higher adherence to MedDiet with lower incidence of CVD [15,90,91,93,94]. Two meta-analyses showed separately an estimate of 10% reduction in fatal or nonfatal incidence of CVD events for each 2-point increase in the score of adherence to MedDiet [91,94]. According to an umbrella meta-analysis, there is strong evidence for the association of adherence to MedDiet with reduced risk of all-cause mortality, as well as incident CHD, CVD, myocardial infarction, and diabetes [15]. For what concerns CV risk factors, a substudy of the PREDIMED trial reported a significant reduction in plasma glucose concentrations, lipid profiles, and systolic blood pressure in participants from the intervention group following MedDiet supplemented with olive oil or nuts vs. those following a low-fat diet [117]. Studies evaluating plasma lipid profiles have shown variable results [118], to some extent due to considerable differences in dietary interventions, conducted among older adults or community dwellers, with or without CVD or at high risk of CVD. Inflammatory markers are also considered markers of CV risk [125]. A higher adherence to MedDiet was related to improved inflammatory markers when compared to an emblematic diet rich in carbohydrates and SFA [119]. Even if specific data on olive oil consumption are limited, a meta-analysis of case-control, prospective cohort studies, and the PREDIMED trial examined the specific association between olive oil consumption and the risk of CHD (101,460 participants) or stroke (38,673 participants). The random effects model combining CHD and stroke events showed a significant reduction (RR 0.82; 95% CI 0.70, 0.96) for a 25 g increase in olive oil consumption [126].

MedDiet model includes the option of consuming a light or moderate amount of alcohol (5–25 g/day for women and 10–50 g/day for men), reported to be associated with a lower risk of total mortality, T2D, CHD, stroke, and heart failure [127]. Epidemiological evidence emerged from large prospective studies nearly three decades ago to support the hypothesis of an inverse association between alcohol consumption and CHD [128]. Evidence for cardio-protective effects of wine had emerged even earlier [129]. Nevertheless, the hypothesis that alcohol protects against CVD has been a subject of debate [130,131,132]; recent data reported an inverse association of alcohol consumption with CHD but an increased risk of different types of stroke [131]. From a public health point of view, it is clear that heavy drinking (more than four drinks per day) represents a risk factor for CVD and other chronic diseases, as well as a relevant disease burden [133]. Alcohol consumption in excess is a leading cause of premature deaths in the USA [134]. Recent studies based on Mendelian randomization analyses in mega-cohorts have questioned the CV benefits of alcohol consumption [132], supporting the public health message that there is no safe level of alcohol consumption. Hence, it is not advisable to begin drinking or drink more often in an attempt to obtain eventual health benefits, as moderate alcohol intake is as well linked to an increased risk of drowning, violence, and injuries from car accidents and falls, and with a higher risk of breast cancer.

However, for wine there still seems to be specific supporting evidence, in particular for red wine [135], which is the main source of alcohol in Mediterranean countries, mostly consumed during meals in moderate amounts [33], and indicated as one of the key contributors to the inverse association of adherence to the MedDiet and all-cause mortality [136]. The French paradox, i.e., the observation of a low incidence of CHD despite a high consumption of SFA was specifically attributed to the high consumption of wine [137]; this notion started a series of investigations on wine and its components. Furthermore, it has been suggested that the composition of red wine, which comprises not only alcohol but also different bioactive compounds (polyphenols) can impact oxidative stress and chronic inflammation highlighting the possibility that a balance between these components may explain its cardioprotective effects [135].

In fact, there are studies showing how the fraction of phenolic compounds in red wine can inhibit LDL oxidation, proposed as a mechanism for delaying the onset of atherosclerosis and decreasing CV risk [138]. This would be confirmed by studies showing that these properties are maintained in dealcoholized wine. A study by Serafini et al. in patients with CHD showed that dealcoholized wine, but not white wine, significantly increased the antioxidant capacity. These effects were attributed to the polyphenol content, which is much higher in red vs. white wine [139]. Another small study showed how the consumption of purple grape juice (without alcohol) was associated with an improvement in endothelial function measured as flow-mediated vasodilation of the brachial artery, which was related to a reduction in the susceptibility of LDL oxidation in 15 CHD patients [140]. An in vitro study with umbilical vein endothelial cells showed that dealcoholized wine significantly increased nitric oxide (NO) production and NO synthase [141]. Dealcoholized red wine has also been reported to have antithrombotic effects in experimental animals through NO-dependent mechanisms [142]. In humans, acute administration of alcohol-free red wine reduced arterial stiffness in patients with CHD, which was attributed to antioxidants content in red wine [143].

Overall, the available evidence hitherto suggests favorable effects of a higher adherence to MedDiet on primary and secondary CVD prevention. Nevertheless, further intervention studies are warranted to validate these encouraging findings.

### 6.2. Cognitive Decline, Dementia, and Depression

Some of the most concerning NCDs, because they are primary causes of disability, are age-associated neurodegenerative diseases including Alzheimer’s (AD), Parkinson’s (PD), and other forms of cognitive decline extending from mild cognitive impairment to vascular dementia. Dementia is currently considered a global epidemic involving over 47 million people, with prospective estimates to almost triple by 2050. About 70% of dementia cases in seniors correspond to AD. Population aging is the primary driver of the projected dementia cases growth. The good news is that about 30% of cases have been linked to probable modifiable factors, including diabetes, midlife obesity and hypertension, smoking, cognitive and physical inactivity, depression, and/or low educational achievement [2,144].

A comprehensive review evaluated the potential effects of specific dietary patterns on cognitive decline and dementia prevention. Dietary patterns with the strongest evidence for slowing cognitive decline, AD, and other dementias, were MedDiet, Dietary Approaches to Stop Hypertension (DASH), and MIND diet combining both MedDiet and DASH (MedDiet-DASH Intervention for Neurodegenerative Delay—MIND) [145]. Several dietary components and supplements have been studied aiming to evaluate whether their potential antioxidant, anti-inflammatory, and vasodilating effects may modify cognitive decline. This includes vitamins (e.g., vitamins D, E, C, beta-carotene, B12, B6, folic acid), omega-3 fatty acids, minerals (e.g., zinc, magnesium), and other nutraceuticals (e.g., curcumoids, acetyl-L-carnitine, gingko biloba, tea, epigallocatechin-3-gallate, caffeine, phytoestrogens, garlic, resveratrol). Overall, all the screened small and large-scale trials with heterogeneous design (i.e., diverse age, confounder adjustments, and exposure time) studying the supplements showed negative or mixed effects. Therefore, they do not confirm a definitive role for these supplements as preventive agents for cognitive decline [113]. As opposed to single components (foods or nutrients), healthy cardio-protective dietary patterns integrating diverse healthy foods/nutrients, such as the MedDiet, has moderately significant evidence of being similarly protective for cognitive deterioration, AD, and PD [90,114,145]. A recent study showed that the MedDiet was associated with lower likelihood of prodromal PD, which confirms the potential actions to delay the onset or lower incident PD [146]. In fact, due to likely synergistic effects of diverse healthy foods and nutrients that characterize a dietary pattern, it is plausible that this complex arrangement can have stronger actions related to neurodegeneration than single nutritional components. MedDiet is full of foods/nutrients with anti-inflammatory and antioxidant properties, which are potentially neuroprotective and that may be considered an optimal nutritional opportunity for the maintenance of brain health.

A systematic review and meta-analysis of prospective cohort studies investigating the effects of MedDiet on cognition outcomes [114] found that a higher adherence to this dietary model was associated with significant reduction in the incidence of AD and cognitive decline. Among cohorts from the USA, Australia, and France, those participants in the highest tertial of adherence to MedDiet had 33% lower risk of developing cognitive decline or AD vs. participants in the lowest tertial of adherence. Although results from interventional studies are still scarce, analyses from the PREDIMED trial are promising. A sub-analysis of data from the PREDIMED trial [115] showed that MedDiet supplemented with either EVOO or nuts was associated with improvements in cognitive function vs. low-fat diet. The MedDiet comprises not only nutritional factors but also lifestyle characteristics, such as social engagement, physical activity, adequate rest, and culinary activities, in addition to the nutritional composition. Some of such lifestyle factors were reported to positively impact the postponement of cognitive decline, beyond diet. Therefore, future studies need to include all the features of Mediterranean lifestyle into their design. Although some studies suggested that adherence to the MedDiet is associated with AD risk reduction, alone or in combination with DASH (MIND diet) [145], there are also inconsistent results, mostly due to substantial heterogeneity regarding methods used for assessing diet and cognition as well as the inclusion of diverse populations. Further validation in large population studies with prospective design and RCTs with lengthier follow-up, among populations from diverse ethnicities and different dietary habits is necessary.

Although evidence for the benefit of adherence to MedDiet on the incidence of depression is scarce, there are some promising results. A systematic review and meta-analysis of 21 studies showed that a high intake of vegetables, whole grains, fruit and fish, which are staples of MedDiet, may be related to a reduction in the risk of depression [88,116]. However, further RCTs with high-quality design as well as prospective cohort studies are still needed to validate this finding.

### 6.3. Cancer

At present, there is agreement as to the notion that various forms of cancer can be prevented, while diet seems to play an essential role in this prevention. A meta-analysis including over 1.7 million participants concluded that a higher adherence to MedDiet was associated with a significantly lower risk of all-cause mortality, breast cancer, several gastrointestinal cancers (including liver and pancreas), head and neck cancer, prostate cancer, and pulmonary cancer [105]. These results, which were derived from observational studies, have been confirmed by data from PREDIMED trial, in which the highest category of nuts consumption, typical component of MedDiet, was associated with a decrease of 40% in cancer mortality when compared with the lowest category [106]. Additionally the extensive European Prospective Investigation into Cancer and Nutrition (EPIC) study has reported data on the MedDiet benefits against incident cancer. The evidence of protection was stronger for colorectal, gastric, and breast cancer, in particular after exclusion of alcohol from the score [107].

Breast cancer has increased by over 20 percent worldwide since 2008 and it is the leading cause of female cancer. Data from the PREDIMED trial reported that after a median 4.8 years of follow-up, the observed rates of breast cancer were lower for the intervention groups with supplemented EVOO or nuts (1.1 and 1.8, respectively) when compared to the control low-fat diet (2.9). After multivariate adjustments, the group receiving the MedDiet supplemented with EVOO had a significant lower risk of developing breast cancer vs. the control group; for each additional 5% calories from EVOO the risk was 28% less [147]. A case-control study involving 2396 Asian American women aged 25–74 years found that the MedDiet was associated with a breast cancer risk reduction of 35% [108]. The Four-Corners Breast Cancer study showed that Hispanic and non-Hispanic women who adopted the MedDiet had a reduced risk of breast cancer [109]. Two prospective studies comprising 91,779 American women [110] and 65,374 French women [111] confirmed a protective association of adherence to MedDiet and the incidence of breast cancer. The protective actions of MedDiet on breast cancer risk have been linked to the reduction in circulating estrogenic levels and to the increased intake of carotenoids, which are known antioxidants lowering oxidative stress. The protective associations were higher in women with progesterone and estrogenic receptor-negative disease.

A lower risk of colon cancer has been related to dietary patterns that are higher in vegetables, legumes, fruit, whole grains, fish, lean meats, low-fat dairy products, with moderate consumption of alcohol, as well as with reduced consumption of red and/or processed meats, sugar-sweetened beverages, and saturated fat. On the contrary, diets that include higher amounts of red/processed meats, sugars (i.e., deserts, sugar-sweetened beverages, and sweets), potatoes and French fries, are associated with a higher risk of colorectal cancer. Data derived from the Italian EPIC study including 42,275 participants, aged 25 to 70 years without cancer history at baseline concluded that a greater adherence to MedDiet was associated with an 8 to 11% decrease in the risk of developing colorectal cancer, both in men and women [112]. The protective effects were mostly observed for distal colon and rectal cancer, while it was less present for proximal colon cancer. There is strong evidence showing that a higher intake of fiber (~30 g/d) is associated with reduced risk of developing insulin resistance and colon adenomas. The main sources of fiber in MedDiet are vegetables, unrefined cereals, legumes, and fruit. Fiber fermentation by the gut microbiota produces protective SCFA acetate, butyrate, and propionate.

A recent systematic review of the associations between adherence to the MedDiet and cancer identified 117 studies up to April 2020 including 3,202,496 participants that were included in a meta-analysis. The results showed that the highest adherence to the MedDiet was significantly associated with lower cancer mortality, lower all-cause mortality among cancer survivors, as well as lower incident breast, colorectal, head and neck, respiratory, gastric, bladder, and liver cancer. Adherence to the MedDiet did not modify the risk of blood, esophageal, pancreatic, and prostate cancer risk [148].

In summary, all the above results suggest that adherence to MedDiet may contribute to the reduction of several forms of cancer and also to the overall cancer-related mortality. Nevertheless, more research is needed regarding the most effective foods and nutrients for this outcome.

### 6.4. Obesity, Metabolic Syndrome, T2D

Obesity, a major health problem with a prevalence that tripled since the 1970s, affects a large portion of the world population across all ages [149]. Overweight and obesity are important risk factors for the development of metabolic syndrome and T2D. A key factor that can help explain the beneficial effects of the MedDiet is the reduction of body weight and adiposity, especially at the abdominal level. Results from the SUN longitudinal project found that participants with the greatest scores of adherence to the MedDiet had a reduced weight gain yearly vs. participants with the lowest adherence [96]. Results from the EPIC-Spain study showed that the highest tertile of adherence to the MedDiet was associated with a lower incidence of obesity and overweight during follow-up [97]. Likewise, participants in the EPIC-PANACEA study, with higher adherence to the MedDiet gained a lower amount of weight in five years vs. participants with lower adherence, and a 10% lower risk of becoming overweight or obese [98]. In this study, a low consumption of meat was the component with most effects against weight gain. In the EPIC-Italy study, an increased MedDiet-index, which assessed conformity with this dietary pattern, was associated with a risk reduction of becoming overweight or obese, with a lower risk of developing abdominal obesity [150]. A meta-analysis of 16 RCTs from Spain, the USA, Israel, Italy, France, Germany, the Netherlands, and Greece including 3436 participants, showed that persons allocated to the MedDiet had significant weight reduction and lower body mass index vs. those in the control group, using a random effects model [99]. The effect of the MedDiet on body weight modifications was larger if associated with increased physical activity, energy restriction, and in participants who were followed for six months or longer. The MedDiet was not associated with weight gain across all 16 studies.

Although the metabolic syndrome definition is still largely debated, there is general agreement on the notion that the concurrent presence of CV risk factors (i.e., hyperglycemia, hypertension, abdominal obesity, and dyslipidaemia) increases the risk of developing CVD and T2D. Recent analyses indicate that aspects defining the metabolic syndrome are among the ten largest determinants of global deaths and disability-adjusted life-years (DALYs) [151]. Therefore, interventions aimed at lowering the risk factors for metabolic syndrome should be included in a public health worldwide strategy. In this context, MedDiet represents an intervention that could help against overweight and obesity, and their negative health consequences. A higher conformance with MedDiet has been associated with the reduction of several cardio-metabolic risk factors in both observational and intervention studies [91,100].

There is strong evidence supporting the notion that dietary modifications decrease the incidence of T2D. Several healthy foods and nutrients, some included in MedDiet, have been inversely associated with incident T2D [152]. Both observational and intervention studies evaluated MedDiet effects on the incidence of T2D [101,102,103,104]. Results from the SUN prospective project conducted in Spain including 13,380 participants found a significant inverse association between adherence to MedDiet and the risk of incident T2D; every two-point increase in MedDiet score was associated with 35% lower risk of T2D [101]. The EPIC-Greece study including 22,295 participants also showed a significantly lower T2D risk with higher adherence to the MedDiet [102]. Likewise, a substudy (*n* = 3541) from the PREDIMED trial confirmed that adherence to a MedDiet supplemented with EVOO without caloric restriction reduced T2D risk in persons at high CV risk [103]. A meta-analysis including 122,810 participants from nine studies confirmed again that a higher adherence to MedDiet was significantly associated with a lower risk of incident T2D [104]. This association was stronger for studies with a longer follow-up. The PREDIMED-PLUS trial examined the long-term effectiveness of an intensive weight loss lifestyle intervention on primary CV prevention in overweight/obese adults with metabolic syndrome aged 55–75 years. The intervention consisted of an intensive energy-restricted MedDiet, physical activity promotion, and behavioral support vs. a control group. After 12 months, participants from the intervention group lost an average 3.2 kg of body weight vs. 0.7 kg in the control group. Several CV risk factors also improved in the intervention group, including waist circumference, fasting blood glucose, triglycerides, and HDL-cholesterol. In addition, there were reductions in insulin resistance and glycated hemoglobin A1c. Participants with diabetes or prediabetes exhibited improvements in their glycemic control, insulin sensitivity, blood triglycerides, and HDL-cholesterol when compared to the control group [153].

As mentioned in Section 3, a crucial mechanism that can help explain the MedDiet’s beneficial effects is the modifications in gut microbiota, which is now considered fundamental in the interplay of diet and health. This mechanism is particularly relevant for metabolic disorders. In fact, there is growing evidence that the gut microbiota composition may affect obesity and diabetes as it is being increasingly recognized as a key factor connecting genes, environment, and the immune system with a role in homeostasis and energy intake from the diet. A study by Gordon et al. suggested that the microbiota from obese persons specifically increases the energy harvested from the diet, providing extra energy to the host [154]. Dietary components can undergo differences in caloric extraction in function of gut microbiota composition. Interestingly, germ-free mice do not become obese following the administration of a Western diet rich in fat and sugar [155].

A study in overweight participants showed a significant increase in propionic acid production in participants following the MedDiet that was inversely related to various inflammatory cytokines [73]. In a sample of the Obekit cohort, a higher adherence to the MedDiet was associated with increased butyrate-producing taxa [74]. A substudy from the PREDIMED-Plus study showed that weight loss induced by an energy-restricted MedDiet plus physical activity induced changes in gut microbiota with increases in SCFAs producing bacteria [156]. Pisanu et al. showed that in a sample of 23 obese patients a moderately hypocaloric MedDiet reduced body weight accompanied with increase in several Bacteroidetes taxa and a depletion of many Firmicutes taxa after three months of intervention [157]. Another study conducted in participants from the CORDIOPREV study reported that MedDiet or a low-fat diet partially restored the gut microbiome dysbiosis in obese patients with metabolic syndrome [158]. A recent study by Tagliamonte et al. investigated whether plasma concentrations of endocannabinoids and N-acylethanolamines were affected by MedDiet, and explored the associations with gut microbiome, insulin resistance and systematic inflammation in overweight and obese participants. MedDiet modified the endocannabinoid system and increased A. muciniphila abundance in the gut independently of body weight changes. Endocannabinoid tone and microbiome functionality at baseline were associated with individualized responses to MedDiet in ameliorating insulin sensitivity and inflammation [159].

In summary, there is evidence suggesting that adherence to the traditional MedDiet may be helpful in the prevention of abdominal obesity, weight gain, and incidence of T2D. Although some studies so far report promising results, much work is still needed to better define the effects of the MedDiet on the gut microbiome in relation to metabolic diseases.

### 6.5. Chronic Respiratory Diseases

Chronic obstructive pulmonary disease (COPD) is the third leading cause of mortality worldwide, with a prevalence of 10.1% afflicting people from low-income, middle-income, and wealthy countries [160]. COPD is a chronic multifactorial pathology strongly influenced by genetics, environmental and behavioral factors, including physical inactivity and unhealthy diet. In the last two decades, several studies have shown that adherence to MedDiet, mainly composed of vegetables, whole grains, fruit, nuts, and fish is associated with a reduced risk of developing COPD [161,162,163,164]. These studies suggested that the antioxidant compounds content in several components of the traditional MedDiet may help to reduce the oxidative microenvironment in the lung. Furthermore, the anti-inflammatory properties of omega-3 fatty acids, contained in nuts and fish, and other anti-inflammatory phytochemical, might be important, due to the central role played by inflammation in the progression of COPD. A Swedish population-based prospective nested case-control study compared 370 participants with incident COPD with 1432 controls without the disease after a mean of 11.1 years of follow-up. Adherence to the MedDiet was assessed by a modified version of the MedDiet score. After adjustment for co-habiting and education level, participants with an intermediate and with the highest MedDiet scores had a 27% lower risk of developing COPD vs. those with the lowest scores, which remained significant after adjustment for smoking intensity [164].

Processed and ultra-processed foods are not part of the MedDiet. Moreover, this type of food is not recommended. Processed meat is not fully excluded but its consumption as part of MedDiet should be infrequent and in moderate portions (Table 1). The lower consumption of processed meat may contribute to maintain a reduced oxidative stress environment in the lung, as reported by a systematic review and meta-analysis including five studies, showing that each 50 g/week increase in the consumption of processed meat was associated with an 8% increased risk of developing COPD [165].

### 6.6. Bone Health

The available evidence on the MedDiet and bone health is scarce. However, recent studies proposed that the MedDiet, as well as other healthy dietary patterns, could be linked to a reduced hip fracture risk. A Swedish study with analyses of data from two cohorts including 71,333 participants with an average age of 60 years, used a modified score to assess the adherence to MedDiet comprising consumption of fruit and vegetables, whole grains, legumes and nuts, fish, fermented dairy products, and olive/rapeseed oil, moderate intake of alcohol, and low intake of red/processed meat. For each unit of increase in the MedDiet score there was a reduction of 6% in hip fracture rate, after adjusting for confounders. Those participants allocated to the highest quintile of MedDiet adherence showed a reduction of 22% in hip fracture adjusted-risk compared with those in the lowest quintile, for both men and women [166].

The traditional Greek MedDiet includes the consumption of yogurt that lately is increasing in other countries. Fermented milk products such as yogurt may be an optimal food choice for persons at high fragility fractures risk, because in addition to being rich in proteins and calcium, they are good sources of pre- and pro-biotics, which have been proposed to be beneficial in the prevention of fractures [167]. Prebiotics are non-digestible fibers that promote the growth of bacteria (probiotics) present in the large intestine, acting as substrates in their reproduction. A Swedish cohort study included 61,240 women, born between 1914 and 1948, assessing their dietary intake by means of a semi-quantitative frequency FFQ in 1987–1990, and followed them for a mean of 22 years. The results of this study showed that a high consumption of fruit and vegetables (>5 servings/day) combined with the consumption of fermented milk (yogurt or soured milk >2 servings/day) was associated with the highest reduction in hip fracture observed (reduction of 19% compared to low consumption of fruit/vegetables plus fermented milk). The authors also found that a high consumption of milk (>3 glasses/day) in combination with a low consumption of fruit and vegetables (<2 servings/day) was associated with a 2.5 fold higher adjusted-risk of hip fractures when compared to a low intake of milk (<1 glass/day) plus a high intake of fruit and vegetables (>5 servings/day) [168]. This is in line with the current notion of considering the benefits from combinations of foods more than from foods and nutrients in isolation. Indeed, one of the best examples of this paradigm is the MedDiet model.

The incidence of fragility fractures is heterogeneous among European countries, but overall it has been reported that it is lower in the Mediterranean area vs. Northern Europe [169]. Several studies reported that the incidence of fragility fractures is lower in persons with a diet more adherent to the MedDiet pattern, including: EPIC-Elderly study [170], EPIC [169], Women’s Health Initiative [171], The CHANCES project [172], and two studies from the Cohort Study of Swedish Men and Women [166,173].

Nevertheless, until now a direct cause-and-effect association between adherence to the MedDiet and a reduction in incident fragility fractures has not been demonstrated. A systematic review and meta-analysis suggested that greater adherence to the MedDiet is indeed associated with a reduced total fracture risk and higher bone mineral density but only three studies were included [174]. Certainly, more studies are needed to make definitive conclusions.

The lack of enough data may contribute to the contradictory observations. As such, another review concluded that there is still limited availability of observational studies supporting a beneficial effect of adherence to the MedDiet and Mediterranean lifestyle on the incidence of hip fractures [175]. However, the interest on nutritional factors that may contribute to the risk of fragility fractures is growing. A longitudinal cohort study examined the degree of adherence to MedDiet in relation to the incidence of fractures among 140,775 participants from five European and US cohorts. The results showed that the incidence of hip fracture was lower in those with medium and high adherence to MedDiet when compared with those with low adherence [176].

There is evidence supporting the concept that some typical components of the MedDiet, such as fruit, vegetables, fish, and low-fat dairy products, are essential for maintaining good skeletal health [177,178,179,180]. Few studies have been performed on humans specifically exploring the effects of MedDiet on fragility fractures. A cohort study nested in the PREDIMED trial evaluated 870 participants randomized to a MedDiet supplemented with extra-virgin olive oil, a MedDiet supplemented with nuts, or a low-fat diet. During a mean follow-up of 8.9 years, 114 incident fragility fractures were documented. In the multivariate-adjusted analyses, participants in the highest tertial of EVOO consumption had a 51% lower risk of fragility fractures compared to those in the lowest tertial [181]. In support of the potential protective skeletal effects of EVOO, an in vitro study showed that EVOO phenolic compounds were able to modulate the cellular proliferation and maturation of osteoblasts, increasing the activity of alkaline phosphatase and stimulating the deposit of calcium in the extracellular matrix [182].

### 6.7. Mortality

One of the most studied outcomes regarding MedDiet is its association with prolonged survival, which has been evaluated in several populations. Afterwards the earlier ecological evidence from the Seven Countries Study [24], the first longitudinal cohort prospective investigations exploring mortality as outcome involved small samples from Greece [49,77], Denmark [84], Australia [85], and Spain [86].

A first study conducted in Greek villages in 1995 collected data from 182 older adults [77] using a validated, extensive FFQ to assess dietary components. An increase of one unit in the MedDiet score was associated with an all-cause mortality significant reduction of 17%. The study conducted in Denmark [84] in order to test MedDiet in a non-Mediterranean population, recruited 202 men and women who were born between 1914 and 1918, and were followed for 6 years. This study used the same original MedDiet score as the Greek study (Trichopoulou’s score), and showed similar results in a Danish population, i.e., for each one unit increase in the adherence to MedDiet score there was an associated significant mortality reduction of 21%. The study conducted in Melbourne, Australia [85], enrolled Anglo-Celts and Greek-Australians older adults, with the purpose of examining whether the MedDiet benefits reported in Greeks and Danish participants may well be extended to populations with very different dietary habits. This study comprised 141 Anglo-Celts and 189 Greek-Australians, men and women, aged more than 70 years. The results were again similar: one unit increase in adherence to MedDiet score corresponded to a 17% significant reduced risk of all-cause mortality. The mortality reduction in relation to adherence to the MedDiet score was equally evident in Anglo-Celts and Greek-Australians. Another study was conducted in Spain [86] involving 161 non-smoker older adults of both genders living in long-term facilities, who were followed for 9 years. The mortality reduction associated with one unit increase in adherence to the MedDiet score in this frail population was even higher (31%), possibly corresponding to a higher mortality risk in this population. However, the effect was significant only for participants aged below 80 years.

A pivotal study was published in 2003 by Trichopoulou et al. [78], which involved 22,043 Greek community dweller adults using a validated FFQ at baseline, with a follow-up of 44 months. There was a significant reduction in all-cause mortality for participants with a higher adherence to MedDiet; for each two-point increase in the adherence score adjusted for confounders there was an associated 25% reduction in overall mortality. Regarding specific mortality causes, the one linked to CHD was reduced by 33%, while cancer-linked mortality was 24% lower for each two-point increase in the MedDiet score.

A multinational European study involving 3496 participants aged 70–90 years from 10 European countries, the Healthy Aging a Longitudinal Study in Europe (HALE) [87], showed that participants who were more adherent to MedDiet had over 50% reduction in all-cause mortality, and mortality related to CVD and cancer. An extensive study conducted in the US [88] corroborated the association of a higher adherence to MedDiet with a reduced mortality, assessing adherence with the original Trichopoulou’s 9-point score. This study enrolled 166,012 women and 214,284 men from the National Institutes of Health-AARP (formerly known as the American Association of Retired Persons) Diet and Health Study. A higher conformity with MedDiet was significantly associated with reduced all-cause deaths and with a significantly lower cause-specific mortality. Specifically, total, CVD, and cancer-related mortality were lowered by 21%, 22%, and 17%, respectively, in men; the decreased risk ranged from 12% for cancer-related mortality to 20% for total mortality in women. After restricting the analyses to participants who had never been smokers the results remained unchanged. A study conducted in Sweden including 1037 older adults, men and women, showed no association of diet macronutrient composition with incident mortality; conversely, a score of MedDiet with some modifications exhibited a significant inverse association of a higher score with reduction in total mortality [183].

Recently, a meta-analysis including data from seven longitudinal cohort studies with 11,738 participants and 3874 deaths, reported a 5% lower risk of total mortality incidence for each one point increase in MedDiet score [89]. Another recent meta-analysis of 30 prospective cohort studies confirmed the inverse association of adherence to MedDiet and all-cause mortality; comparing highest vs. lowest adherence the risk was reduced by 21% in the highest group. As regards MedDiet components, relatively stronger inverse associations were found for moderate/none-excessive alcohol consumption and for fruit and vegetable consumptions, whereas a positive association was apparent for meat consumption [184].

In agreement with the evidence previously described, new research is exploring whether combining the effects of a MedDiet-like pattern with regular physical activity can further reduce all-cause mortality. A large prospective study of the SUN cohort, analyzing data from 19,467 participants, showed that the MedDiet and physical activity were associated with significant reduction in overall mortality risk [185]. Another prospective study, involving 7356 older adults with high CV and a mean age of 67 ± 6.2 years, showed that strict adherence to the MedDiet and higher levels of leisure-time physical activity, regardless of the intensity, were both separately and together associated with reduced all-cause mortality [186]. Likewise, analyses from the Melbourne Collaborative Cohort Study, which followed 22,213 participants for 13.6 years, showed that the combination of adherence to a MedDiet and high physical activity resulted in an estimated reduction of 1.82 per 100 people in all-cause mortality. The population attributable fraction was 13% for sustained high physical activity, 7% for sustained adherence to a MedDiet, and 18% for their combination [187].

To summarize, several studies in diverse populations provide evidence for the beneficial effects of the MedDiet pattern on the risk of all-cause deaths, and on the risk of CV- and cancer-related deaths (also discussed in specific sections above). This highlights the relevance of implementing or maintaining the use of this dietary model in order to maximize the probability of survival.

## 7. Sustainability

The seminal report from the EAT-Lancet Commission on healthy diets from sustainable food systems highlights the evidence that a diet rich in plant-based foods and fewer foods of animal origin confers not only optimal health outcomes but also environmental benefits [12]. The Commission presented an integrated framework providing scientific targets for healthy diets and sustainable food production. It estimates that the transformation to healthy diets by 2050 will require that the world’s consumption of fruit, vegetables, nuts, seeds, and legumes doubles, and the consumption of foods such as red meat and sugar should be reduced by more than 50%. The report describes a universal healthy reference diet that provides a basis for estimating the human health and environmental effects of adopting an alternative diet to current diets, which are frequently full of unhealthy foods that cause micronutrient deficiencies and contribute the considerable upsurge of diet-related obesity and diet-related NCDs, including CVD, stroke, and diabetes. The report states that the best studied example of such diet is the MedDiet, similar to the diet of Crete in the mid-20th century. It also warns that if nothing is done, today’s children will inherit a planet that has been severely degraded and where a large part of the population will suffer more from malnutrition and preventable diseases.

In fact, the traditional MedDiet, moreover its CVD and cancer beneficial actions traditionally documented, has various other benefits, which are areas of current new research, as mentioned in the previous sections. Furthermore, the notion of the MedDiet has lately evolved from being considered a healthy dietary model to be recognized as a sustainable eating pattern, in which nutrition/food, physical activity, social engagement, traditions, gastronomy, cultures, agriculture, environment, and sustainability interact to conform an archetype of an ecologically friendly healthy way of eating and living. Analyses of data from the Spanish SUN project comparing the environmental footprints and monetary costs of MedDiet, Provegetarian, and Western dietary patterns in a scale of harmful environmental effects found that Provegetarian and MedDiet scored significantly better compared to Western diets, which were the most detrimental [188].

Aside from its rising attractiveness worldwide, adherence to MedDiet is being abandoned, even in countries located in the Mediterranean region where the first beneficial effects were observed [21,22,23,189]. This is probably due to several reasons, particularly related to the influence of the Western economy, with globalization of food production and consumption, as well as spreading of urbanization, technology, and tourism. The paradigm of healthy eating, of which the MedDiet is a primary example, is in serious threat. Factors that notably contribute to this hazard include: (i) the propagation of the consumption of fast-food, centered on meats, potatoes, refined cereals, sweets, ice cream, and sugar-sweetened drinks; (ii) the occurrence of economic crisis, which makes it necessary for the most underprivileged populations to choose low-cost industrial food, full of empty calories but poor in nutrients; (iii) the popularity of high-protein low-carbohydrate diets, plenty of meat products, used with the purpose of losing or maintaining body weight, with an extensive impact on human health and on planet protection. Such events represent wide-ranging dangers for the conservation and transmission of the intangible MedDiet heritage for the forthcoming generations. Adherence to the MedDiet has strong socioeconomic connotations, as shown by analyses of data from the MOLI-SANI study reporting that MedDiet is independently more followed by people of higher income and education level and with a lower prevalence of obesity [190]. These inequalities seem to have been accentuated since 2007, the year in which the economic crisis began and wealth became an always more relevant determinant of adherence to MedDiet, as opposed to previous years [189]. A paradox, considering that at the time of Ancel Keys in the 1950s, MedDiet was indeed the diet of the farmers, of those who had no ways to buy food. Now the scenario has certainly changed, being the wealthy health-conscious populations, those who mostly follow the MedDiet.

Thus, it is essential that society and governments commit to develop programs aiming to preserve the precious knowledge of the MedDiet based on cultural and traditional foundations, encouraging the communities towards sustainable diets and food diversity, which at the same time offer short and long-term health outcomes as well as environmental benefits.

## 8. Extrapolation of the MedDiet to Non-Mediterranean Nations

The MedDiet as a paradigm of healthy eating and lifestyle has aroused great interest worldwide. As a consequence, there are currently growing numbers of studies that apply the principles of the MedDiet in epidemiological and clinical investigations as well as in nutritional guidelines outside the Mediterranean region. As already pointed out in the previous sections of this review, the associations of adherence to the MedDiet with different health outcomes have been evaluated not only in Mediterranean countries but also in diverse non-Mediterranean contexts, for example mortality [84,85,88,183,187], cancer [108,109,110], COPD [164], fragility fractures [166,171], and the various systematic reviews, meta-analyses, and multicenter studies already cited included both Mediterranean and non-Mediterranean populations. MedDiet or MedDiet-like diets have been also recommended in national guidelines such as the 2015–2020 Dietary Guidelines for Americans [18,19], American Heart Association (AHA) Guidelines for the prevention of stroke and transient ischemic attack [191], AHA Guidelines on the Primary Prevention of CVD [192] and incorporating MedDiet in healthy eating guidelines in other countries, such as Australia [32] and Ireland [193], have been proposed. However, translating the MedDiet adherence-associated benefits into non-Mediterranean countries can be challenging, particularly because the definition of MedDiet and the methods used to measuring adherence to this dietary pattern as well as the study designs and involved populations are heterogeneous. In addition, there are several misconceptions and difficulties to interpret the recommendations and extrapolate the principles of MedDiet into other contexts, especially because available food composition, type, variety, and method of preparation may influence the evaluation of the effectiveness. A relevant barrier is the fact that olive oil, a cornerstone of MedDiet, is not yet widely used as a main source of fat. Because MUFA content of olive oil is considered to be one of its beneficial components, MUFA:SFA ratio has been used as an indicator of adherence to MedDiet in non-Mediterranean countries. However, MUFA may reflect the consumption of animal fat in populations not consuming olive oil frequently [122]. Furthermore, phenolic compounds contained in EVOO have been hypothesized to be cardioprotective, regardless of MUFA content [194]. As mentioned above, the beneficial effects of red wine consumption in low to moderate amount have been attributed to bioactive compounds contained in red wine and the low level of alcohol intake [135]. However, the evaluation of alcohol intake may not accurately discriminate binge drinkers consuming large amounts of spirits, from frequent consumers of red wine in low quantities. These difficulties and limitations do not completely prevent the extrapolation of the benefits of the MedDiet in other contexts, with due adaptations to local characteristics, not only of the diet and available foods, but also of culture and traditions. For example, it has been reported that some Scandinavian populations are more adherent to the MedDiet than Mediterranean populations [195]. Further research on the transferability and effectiveness of the MedDiet in non-Mediterranean populations and its health benefits is needed.

## 9. Conclusions

The MedDiet model meets various primary criteria for considering it a high-quality healthy diet, among various healthy eating patterns. From the beginning of its conceptualization at the time when Ancel Keys started his studies up to now, convincing evidence has accrued over the years indicating that the traditional MedDiet may play a valuable positive role for health and longevity. Several other patterns have been studied, but still none is supported by such steady prospective observational and trial-based evidence confirming the reduced mortality rate and lower incidence of several NCDs associated with aging. On the other hand, poor-quality diet is now a primary amendable cause of death and disability worldwide.

The effects of MedDiet on the prevention and treatment of CVD are well-recognized. Numerous studies confirm the positive effects of following MedDiet on metabolic disorders and in some types of cancer. Although the evidence is still scarce, existing results indicate that MedDiet may play a preventive role in the development of neurodegenerative diseases, respiratory chronic diseases, hip fractures, and depression. Yet, additional validation in well-designed prospective studies and RCTs with lengthier follow-up, conducted in population with dissimilar dietary habits and from different ethnicities is still needed. Nutritional research is emphasizing the importance of studying dietary patterns rather than individual nutrients or foods with the purpose of identifying possible synergistic, antagonistic, and nutrients/foods combinations actions and substitutions. For example, mixed eating models with both beneficial and harmful components may result in a general null effect. The MedDiet model is not only an optimal combination of nutritional elements, but also a dietary pattern that culturally and historically goes together with other lifestyle healthy factors, such as social engagement, physical exercise, palatable eating, and adequate rest, respecting traditions that have been transmitted from generation to generation for a long historical time. Furthermore, research evaluating the impact on the environment associated with food patterns have concluded that a shift from animal-based diets towards plant-based diets, such as MedDiet, are more environmentally friendly, therefore, more sustainable.

**Figure 3:**
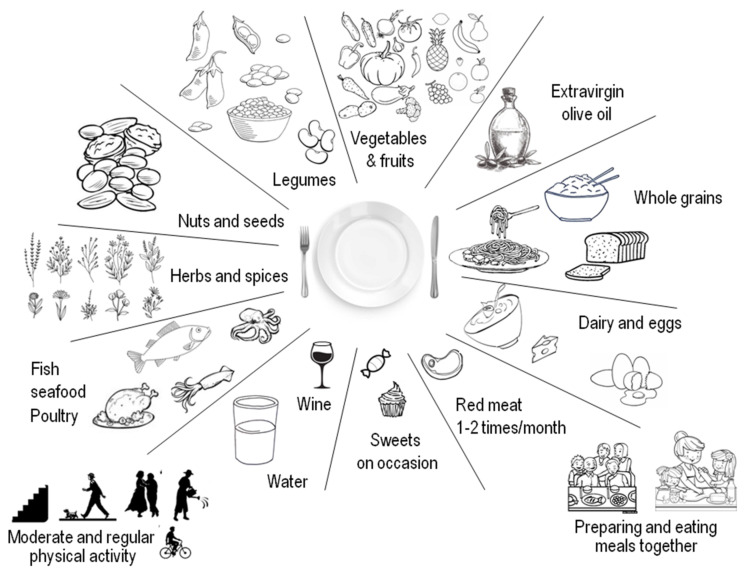
Nutritional and lifestyle components of the MedDiet, which has been associated with improved longevity and reduced incident age-related chronic NCDs.

Unfortunately, Mediterranean countries are abandoning this invaluable legacy. A current concern to consider in order to implement healthy and sustainable eating models worldwide regards the misleading advertising of foods and nutrients, which particularly target children and adolescents through television, internet, product packaging, social media, and other similar means. It has been shown that nearly all foods featured in advertising targeting young people have high content of calories, saturated fat, sugar and sodium [196]. Furthermore, claims about health and nutrition have been found in over half of non-essential foods [197], and parents frequently misinterpret the meaning of front-of-package claims, especially when placed on products with high levels of nutrients to limit (e.g., sugar, sodium) and low levels of nutrients to encourage (e.g., fiber, protein) [198]. The remarkable rise in childhood obesity has urged calls for policy actions to control the marketing of unhealthy food to children, but food industry groups claim that the commercial speech doctrine allows them to openly and legally target these products to young people [199]. However, cognitive research indicates that young children and adolescents cannot effectively recognize the convincing intent of advertising or do a critical evaluation required to understand commercial messages [200]. International continuous actions should be initiated by policymakers and by the general public aiming to protect the intangible legacy of traditional knowledge and health of the Mediterranean way of living, and endorse its propagation worldwide, according to eating traditions and lifestyle of each region, while bearing in mind lifelong and person-tailored approaches. An easy language, probably more visual, should also be sought to direct the population to choose healthy foods and behaviors (Figure 3). Some powerful intrinsic Western socio-cultural values, norms and traditions may represent challenging obstacles to cope with the purpose of implementing the MedDiet or MedDiet-like patterns globally.

## Figures and Tables

**Table 1:** Traditional MedDiet and Lifestyle Features.

• Daily consumption of various fresh vegetables and fruit; nuts, seeds.
• Grain products (bread, pasta, rice), mostly whole.
• Consumption of legumes several times per week.
• Cold pressed extra virgin olive oil for cooking and for seasoning as the main source of fat.
• Herbs and spices, adding flavor to dishes.
• Fresh fruit daily as dessert; infrequent consumption of sweets, cakes, and dairy desserts.
• Fish and seafood (2 to 3 times weekly).
• Daily consumption of dairy, in particular yogurt (small portions of cheese less frequently).
• Eggs, source of high-quality proteins, 2 to 4 times weekly.
• Infrequent consumption of red/processed meat, in small portions (1 to 2 times per month) *.
• Water as the main beverage.
• Drinking moderate amounts of wine always with meals (for women: ≤1 drink/day; for men: 1 to 2 drinks/day) **.
• Preferring fresh, locally produced foods, which have been minimally processed.
• Connection and respect with nature.
• Flavorsome cooking.
• Moderate portion sizes.
• Moderate physical active every day.
• Preparing and consuming meals in the company of other people.
• Have an appropriate rest (enough time and quality of night-sleep and eventually sleeping for a short period of time during the day if necessary [siesta]).

* preferably as a part of stews and other recipes. ** respecting former habits and beliefs.

**Table 2:** Main phytochemicals contained in fruit and vegetables with bioactive properties.

Phytosterols	Organosulfur Compounds	Carotenoids	Alkaloids	Phenolics
Sitosterol Campesterol Stimasterol Sitostanol Campestanol	Alliin γ-glutamyl-5-allyl-L-cysteine Glucosinolates and derivatives	α-carotene β-carotene β-cryptoxanthin Lutein Zeaxanthin Lycopene	Caffeine Trigonelline	Flavonoids Phenolic acids Lignans Stilbenes Coumarins Tannins

Source: Linus Paulin Institute at https://lpi.oregonstate.edu/mic (accessed on 30 March 2021).

**Table 3:** Main phenolic compounds in fruit and vegetables.

Flavonoids	Phenolic Acids	Lignans	Stilbenes	Tannins
Flavonols Flavan-3-ols Isoflavones Anthocyanidins Flavanones Flavones	Hydroxycinnamic acid derivatives - Caffeic acid - Ferulic acid - Curcumin	Cinnamic acid	Resveratrol	Proanthocyanidins

Source: Linus Paulin Institute at https://lpi.oregonstate.edu/mic (accessed on 30 March 2021).

**Table 4:** Main flavonoids in fruit and vegetables.

Flavonols	Flavan-3-ols	Isoflavones	Anthocyanidins	Flavanones	Flavones
Quercetin Kaempferol Myricetin	Catechin Epicatechin Epigallocatechin Epigallocatechin gallate Epicatechin gallate	Genistein Daidzein Biochanin A	Cyanidin Delphinidin Malvidin Pelargonidin	Hesperetin Naringenin Eriodictyol	Apigenin Luteolin Baicalein

Source: Linus Paulin Institute at https://lpi.oregonstate.edu/mic (accessed on 30 March 2021).

**Table 5:** Two scores evaluating adherence to the MedDiet pattern.

Medas * (0–14 Points) [79,80,81]	Mediterranean Diet Score (0–9 Points) [78]
Olive oil as the main culinary fat ≥4 tablespoons per day of olive oil ≥2 servings per day of vegetables ≥3 servings per day of fruit ≥3 servings per week of legumes ≥3 servings per week of fish ≥3 servings per week of nuts ≥2 servings per week of olive oil sauce with tomato, garlic, and onion (“sofrito”) Preference for poultry more than for red meats <1 serving per day of red/processed meats <1 serving per day of butter/margarine/cream <1 serving per day of carbonated/sugared sodas <2 servings per week of commercial bakery, cakes, biscuits, or pastries ≥7 glasses per week of wine	Vegetables ** Fruit and nuts ** Legumes ** Fish ** Cereals ** MUFA/SFA ratio ** Meat/meat products # Dairy products # Alcohol: 5 to 25 g/day (women) 10 to 50 g/day (men)

* MEDAS: Mediterranean Diet Adherence Screener. ** One point added to the score if the consumption results at or above the sex-specific median for the studied population. # One point added to the score if the consumption results below the sex-specific median for the studied population.

## Data Availability

No new data were created or analyzed in this study. Data sharing is not applicable to this article.
